# WEALTH INEQUALITIES IN PHYSICAL AND COGNITIVE IMPAIRMENTS AMONG OLDER ADULTS ACROSS EUROPE AND JAPAN

**DOI:** 10.1093/geroni/igac059.2089

**Published:** 2022-12-20

**Authors:** Nekehia Quashie, Dung D Le, Yoko Ibuka, Martina Brandt

**Affiliations:** University of Rhode Island, Kingston, Rhode Island, United States; Keio University, Tokyo, Tokyo, Japan; Keio University, Tokyo, Tokyo, Japan; Technische Universität Dortmund, Dortmund, Nordrhein-Westfalen, Germany

## Abstract

Although prior research has provided insights on the association between country-level factors and health inequalities, key research gaps remain. First, most previous studies examine subjective rather than objective health measures. Second, the wealth dimension in health inequalities is understudied. Third, a handful of studies explicitly focus on older adults. To bridge these research gaps, this study aims to: i) measure wealth-related health inequalities in physical and cognitive impairments; and ii) examines the extent to which welfare states moderate wealth inequalities in physical and cognitive impairments among older people across Japan and Europe. We utilized harmonized data of non-institutionalized individuals aged 50-75 for physical and cognitive impairments from the Japanese Study of Aging and Retirement (JSTAR) and the Survey of Health, Ageing and Retirement in Europe (SHARE), for (n= 31,969 and 31,348, respectively). We applied a concentration index to quantify the degree of wealth inequalities in impairments. Our multilevel linear regression analyses examined whether national public health spending and healthcare access resources explained cross-country differences in wealth inequalities in physical and cognitive impairments. Findings indicated that inequalities in both impairment outcomes favored wealthier individuals in all countries, but the magnitude of inequality varied across countries under examination. Furthermore, a higher share of public health spending, lower out-of-pocket expenditures, and higher investment in healthcare resources were associated with lower wealth inequalities especially for physical impairments. Our findings suggests that different health interventions and policies may be needed to mitigate specific impairment inequalities.

